# Role of Ti_3_AlC_2_ MAX phase in regulating biodegradation and improving electrical properties of calcium silicate ceramic for bone repair applications

**DOI:** 10.1038/s41598-024-74859-7

**Published:** 2024-10-28

**Authors:** Rasha A. Youness, Mohammed A. Taha

**Affiliations:** 1 Spectroscopy Department, National Research Centre, El Buhouth St, Dokki, Giza, 12622 Egypt; 2 Solid State Physics Department, National Research Centre, El Buhouth St, Dokki, Giza, 12622 Egypt; 3 Pharos University in Alexandria, Canal Mahmoudiah Street, Smouha, Alexandria, Egypt

**Keywords:** Calcium silicate, Biodegradation, Bioactivity, Electrical conductivity, Bone fracture healing applications, Biophysics, Materials science

## Abstract

Calcium silicate ceramic is a promising bioceramic for various biomedical applications, but its high biodegradation rate and low strength restrict its clinical utility. As a result, the study devised an innovative solution to address these issues by utilizing the titanium aluminum carbide phase, potentially for the first time in biological applications, in conjugation with hydroxyapatite. Then, using powder metallurgy technology, they added these phases to calcium silicate to create nanocomposites. After soaking in simulated body fluid for ten days, the produced nanocomposites were assessed for bioactivity and biodegradability using scanning electron microscopy, inductively coupled plasma-atomic emission spectroscopy, and weight loss assays. Their electrical and dielectric properties were also measured before and after soaking in the simulated body fluid solution. Furthermore, the tribo-mechanical properties of all sintered samples were measured. Interestingly, adding 40% hydroxyapatite nanoparticles to calcium silicate reduced the porosity from 12 to 6%. However, adding five vol% of the titanium aluminum carbide phase to the same sample increased the porosity to 8%. Importantly, these recorded percentages of porosity were comparable to those of compact bone porosity, which range from 5 to 13%. The addition of hydroxyapatite and titanium aluminum carbide phase significantly improved the rapid biodegradation of calcium silicate, albeit with a slight decrease in its bioactive properties, as evidenced by the incomplete surface coverage of the samples with the hydroxyapatite layer in the scanning electron microscopy images. The electrical properties of the nanocomposites were better with the addition of hydroxyapatite and titanium aluminum carbide phase, which helped the bone heal faster. The addition of a titanium aluminum carbide phase significantly improved the mechanical properties of the resulting nanocomposites. For example, the calculated values for compressive strength of all examined samples were 131, 115, 105, 147, and 135 MPa. Based on the results, the prepared samples can be used in orthopaedic and dental applications.

## Introduction

Although bone may naturally regenerate tissue as part of the healing process after an injury, this capacity does not apply if the lesion is larger than the critical size defect. In such situations, numerous studies have shown the use of both autologous and allogeneic transplants. However, studies have found that problems such as scarring, impairment, and donor site damage affect up to 20.6% of autologous transplant cases. During the sterilization and preservation procedure, allograft transplants’ biological and mechanical characteristics change, resulting in a loss of osteogenic and integration capacity. Researchers are developing biomaterials for bone substitutes in response to these drawbacks^1^. Researchers have conducted extensive studies to identify the most suitable biomaterial with exceptional features for these applications. Unfortunately, researchers have yet to develop an ideal biomaterial to enhance bone regeneration. Current research aims to provide new alternatives to synthetic bone that closely resemble or mimic natural bone^2^.

Due to their exceptional bioactivity and biocompatibility, calcium silicate (Ca-Si) bioceramics are considered a novel and advanced material for orthopaedic and dental purposes. The primary constraints impeding their adoption in clinical settings include their rapid breakdown, which raises pH levels in the body and affects their ability to integrate with bone tissue, their lack of support for osteoblast growth, and their poor tribo-mechanical characteristics^3–5^.

Orthopaedic and dental applications often utilize hydroxyapatite (HA) due to its superior properties over other biomaterials. It is thought that adding carbonate (CO_3_)^2−^ groups to the crystal structure of HA would make the resulting A-type or B-type carbonated HA more soluble and helpful in living things. Furthermore, it is essential to note that producing CHA on a nanoscale level leads to enhanced properties^6^. Notably, its slow degradation rate and poor mechanical properties limit its therapeutic applications^7^. To address this significant problem, it is essential to improve its mechanical properties by strengthening it with an additional phase possessing the required mechanical strength.

Two-dimensional nanomaterials have grown exponentially since 2004, attracting interest in energy storage, catalysis, flexible electronics, and triboelectric nanogenerators. In 2011, researchers discovered MXenes as a few-atom-thick layered 2D transition metal carbides, nitrides, and carbonitrides. In MXene single flakes, layers of carbon/nitrogen (X) and transition metals (M: groups 3–6 of the periodic table) are bonded together, and the transition metal surfaces have bonded endings (T_x_: –O_2_, –F_2_, –(OH)_2_, –Cl_2_, or a mix of these). Crystal structures and chemical formulas come from their 3D crystalline progenitors, generally MAX phases. A-group elements (mainly groups 13‒16 of the periodic table) sandwich MXenes’ M-X layers in MAX phases, defined as M_*n*+1_AX_n_ (*n* = 1‒4)^8^. The M represents an early transition metal, A denotes an IIIA- and IVA-group element, and X stands for C and/or N (e.g., Ti_2_GeC, V_2_GeC, Cr_2_GeC, Ti_2_SnC, Ti_2_PbC, Ti_3_AlC_2_, Ti_3_SiC_2_, Ti_3_GeC_2_, Ti_4_AlC_3_, etc.)^9–11^. The Ti_3_AlC_2_ MAX phase is bulky (4.21 g/cm^3^), has a high Young’s modulus (297 GPa), conducts electricity well (3.48 × 10^6^ S/m), and does not expand much when heated (9.2 × 10^−6^ K^−1^)^12^. Based on these fantastic properties, scientists believe that biomaterials enhanced with MAX or MXene could be promising for use as bone substitutes^13^.

Various ways for creating nanocomposites have been developed recently. One of these methods is the powder metallurgy (PM) technique. Utilizing traditional PM is very successful for creating nanocomposites due to its capacity to produce uniformly distributed nanoparticles in the composite creation process. The mechanical alloying method compresses and sinters the powder it makes to a suitable temperature to create dense composites^14^. This study employed this concept to prepare the required nanobiocomposites.

Several researchers have used other ceramic materials^15–17^ or polymers^18–20^ to overcome the inherent constraints of CaSiO_3_, which restrict its broad usage in orthopaedic and dentistry fields. As far as the authors are aware, previous investigations have not used two ceramic materials − HA and advanced ceramics such as Ti_3_AlC_2_ MAX phase − in order to regulate the biodegradability of CaSiO_3_, improve its mechanical, tribological, and electrical properties, and maintain its biological capabilities.

## Materials and methods

### Materials

In this work, calcium carbonate (CaCO_3_; 99.6%), calcium hydrogen phosphate dihydrate (CaHPO_4_⋅2H_2_O; 99.5%), titanium (Ti; 99.5%), aluminum (Al; 99.9%), graphite (C; 99.8%), calcium silicate (CaSiO_3_; 99.5%), sodium chloride (NaCl; 99%), sodium bicarbonate (NaHCO_3_; 99.3%), potassium chloride (KCl; 99.2%), calcium chloride (CaCl_2_; 99.4%), dibasic potassium phosphate (K_2_HPO_4_; 99.4%), magnesium chloride (MgCl_2_; 99%), tris‒hydroxymethyl‒amino‒methane ((CH_2_OH)_3_CNH_3_), and hydrochloric acid (HCl; 99.2%) have been supplied from Sigma-Aldrich.

### Methods

#### Synthesis of HA nanopowders

**Figure 1:**
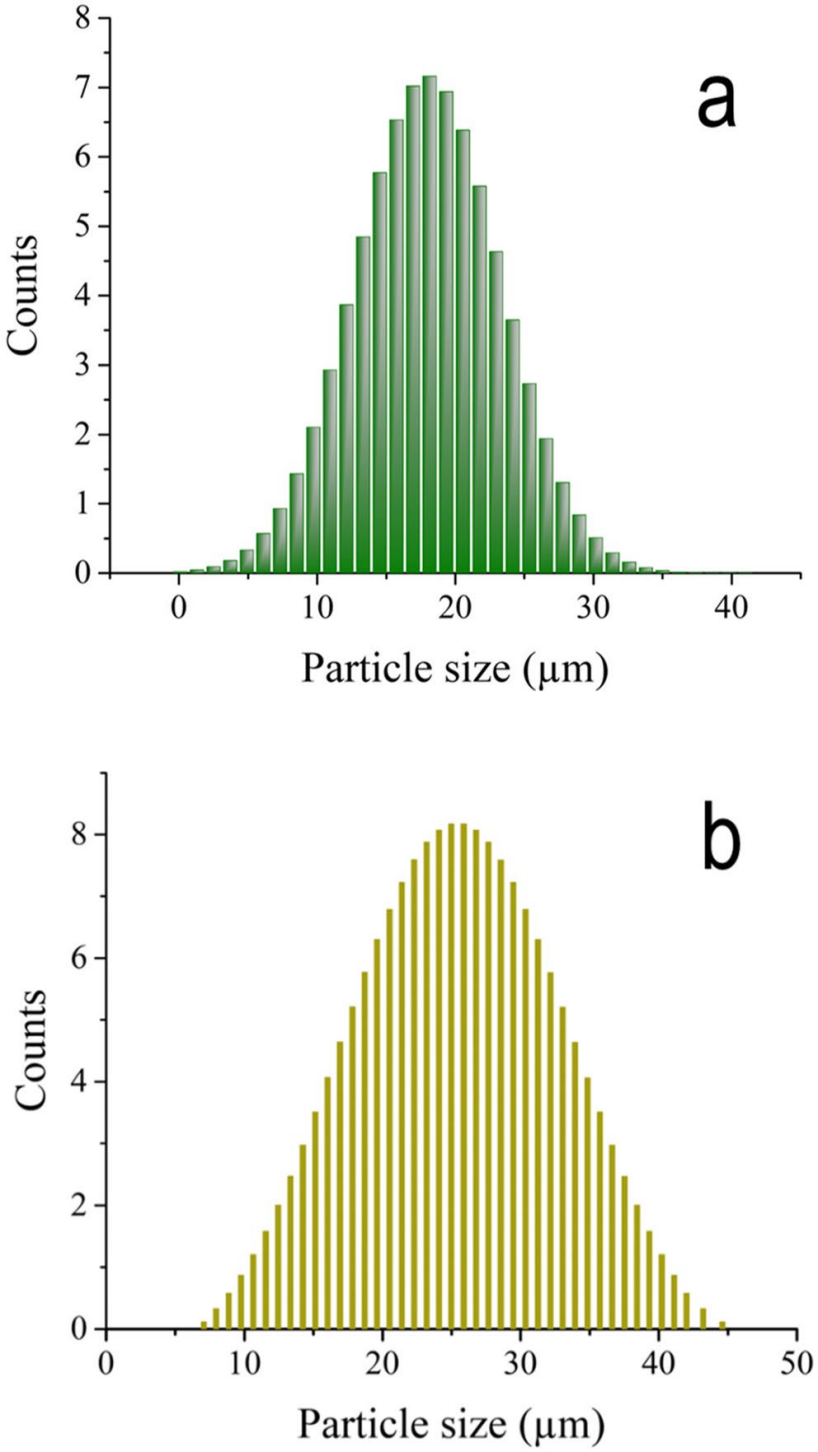
Particle size distribution of as-received (**a**) CaCO_3_ and (**b**) CaHPO_4_.2H_2_O powders.

**Figure 2:**
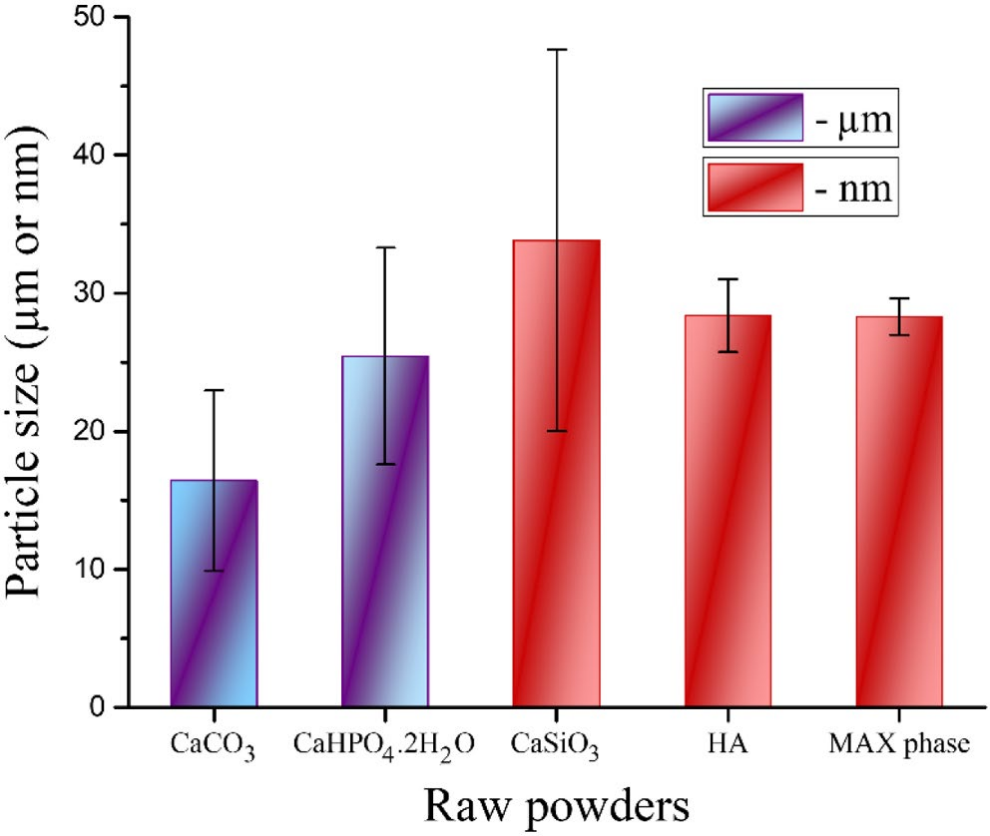
The average particle sizes of the raw materials used, i.e., **a**) CaCO_3_ and **b**) CaHPO_4_.2H_2_O,**c**) CaSiO_3_, **d**) HA, and **e**) Ti_3_AlC_2_ powders.

HA nanopowders were synthesized with partial replacement of some PO_4_^3−^ groups with CO_3_^2−^ groups, similar to the technique reported in our previous work^21^. CaCO_3_ and CaHPO_4_⋅2H_2_O powders with particle sizes ≤ 20 μm and ≤ 30 μm were thoroughly mixed and then processed using a high-energy ball mill (HEBM) to activate the chemical interaction between the raw materials. Laser diffraction particle size was used to determine the particle size, and the distribution pattern of the powders is shown in Fig. 1(a, b) and 2, respectively.

#### Production of Ti3AlC2 MAX phase

Ti, Al, and C powders with particle sizes of 60, 80, and 30 µ, respectively, were used to create the Ti_3_AlC_2_ MAX phase. The ratio of raw materials utilized to produce the MAX phase of Ti: Al: C was 3: 1.2: 2. The powders were milled for 15 h at 350 rpm and had a ball-to-powder ratio (BPR) of 20:1 after being weighed. The powdered materials were subjected to a heat treatment at 1350 °C for 2 h under a vacuum at a heating rate of 3 °C/min.

#### Synthesis of CaSiO_3_/HA/MAX phase nanocomposites

High-resolution transmission electron microscopy coupled with selected area electron diffraction (HRTEM-SAED; JEOL JEM-2100 Japan, operated at accelerating voltage of 120 kV) technique combined with the X-ray diffraction (XRD; Philips PW 1373; X-ray powder diffractometer with CuK-Ni filtered radiation) method was used to analyze the particle size and phase composition of the raw materials, i.e., CaSiO_3_, HA and Ti_3_AlC_2_ powders, respectively. Then, using HEBM to assist, the corresponding volume percentages of CaSiO_3_ (particle size of ~ 40 nm), HA, and MAX phase nanopowders, as shown in Table 1, were mechanically blended in Al_2_O_3_ vials and balls with a diameter of 10 mm at a rotating speed of 150 rpm for 48 h, BPR = 5:1 while operating in a dry situation. The powdered nanocomposites were then sintered at 1050 °C for an hour at a heating rate of 5 °C/min after being compressed using a hydraulic press at 30 MPa.

**Table 1:** Scheme of the prepared nanocomposites, referring to the sample code and its composition (vol%).

Sample name	CaSiO_3_	HA	MAX phase
CHM1	100	0	0
CHM2	80	20	0
CHM3	60	40	0
CHM4	80	20	5
CHM5	60	40	5

#### Characterization of the produced nanocomposites

The XRD method was used to analyze the phase composition of each sintered sample, taking into account that the reference data for the interpretation of the XRD patterns were obtained from ICCD X-ray diffraction card files. FESEM, equipped with energy dispersive X-ray analysis (EDX) (specifically a Quanta FEG250 with an accelerating voltage of 30 kV and a magnification ranging from × 10 up to × 300,000), was used to analyze their microstructure through washing samples with ethyl alcohol, then allowing them to dry and coating them with a thin layer of gold to increase the brightness of the samples.

### Analyzing the produced nanocomposites’ biological properties

#### Bioactivity

The acquired samples were soaked for ten days in an SBF solution prepared according to the instructions of Kokubo et al.^22,23^. , keeping the ratio of sample grains to solution volume = 0.01 g/ml^3^^24^. This allowed the bioactivity of the samples to be evaluated in vitro.

SBF is an aqueous solution devoid of cells with a virtually identical inorganic ion composition to human blood plasma. With a pH of 7.4, SBF is a protein-free solution. According to the concentrations listed in the sources mentioned above, SBF solution was often made by combining the proper quantities of NaCl, NaHCO_3_, KCl, CaCl_2_, K_2_HPO_4_, and MgCl_2_ in deionized water in a beaker with the use of a magnetic stirrer. The mixture was then buffered with (CH_2_OH)_3_CNH_3_ and HCl to a pH of 7.4. In an agate mortar, the materials were crushed into tiny particles, and then they were sieved through various mesh sizes to produce particles with a size range of 106–180 μm. The soaking samples were then subjected to FESEM-EDX examination to determine the surface alterations that had occurred and the change in surface roughness of the soaked samples.

#### Biodegradability examinations

The samples were biodegraded in SBF solution, and weight loss measurements were used to observe the behaviour of the samples. To calculate the percentage of weight loss after an immersion time (t), the following equation was used:

1 $$Weight\;loss\;\left(\%\right)=\frac{\mathrm Wi-\mathrm Wt}{\mathrm Wi}\times100$$

where W_t_ is the weight of the sample disc after a time (t) of soaking in the SBF after it has been dried, and W_i_ is the initial weight of each sample as a disc, i.e., before soaking. Three separate measures of weight reduction were made.

Using the inductive coupled plasma-atomic emission spectroscopy (ICP-AES), the concentrations of Ca, Ti, Al, Si, and phosphorus (P) ions in the SBF solution were determined before and after the samples were soaked in the solution for ten days.

### Analyzing samples properties

#### Physical properties of the investigated samples

The bulk density, relative density, and total porosity of every sintered solid were measured using the Archimedes method (ASTM: B962-13).

#### Mechanical properties

By measuring at least five indentations per specimen for each data point, ASTM B933-09 was used to evaluate the microhardness of the produced specimens. Conversely, each sample’s compressive strength was assessed in accordance with ASTM C1358.

#### Tribological properties

The wear test was conducted using a pin-on-disk tester machine, and the specimens were weighed and measured using a digital scale with a precision of 0.0001 g. The wear test’s process parameters include a speed of 0.8 m/sec and a distance of 480 m. The experiment was conducted with two distinct applied loads, i.e., 20 and 40 N, for 10 min. The tribometer pin and disk were made from RS steel. The wear rate resulting from the loss of weight was determined using the following formulas:

2 $$\mathrm{Net}\;\mathrm{weight}\:=\:\mathrm{Weight}\;\mathrm{before}\;\mathrm{wear}\;-\;\mathrm{weight}\;\mathrm{after}\;\mathrm{wear}$$

3 $$\mathrm{Weight}\;\mathrm{rate}\;=\;\mathrm{Net}\;\mathrm{weight}/\mathrm{time}$$

In addition, the coefficient of friction was also measured using the same method described above.

#### Electrical and dielectric characteristics both before and after soaking in SBF solution

Using a broadband dielectric spectroscopic analysis, the room temperature electrical conductivity, dielectric constant (ε′), and dielectric loss (ε′′) of the generated samples were ascertained. A Novocontrol high-resolution alpha dielectric analyzer operating in the 0.01 Hz–20 MHz frequency range was used to evaluate the dielectric properties. The Quadro temperature controller, which used pure nitrogen as a heating agent and offered temperature stability greater than 0.2 K, supported the analyzer. The measurements were performed at lower and higher frequencies, i.e., 1 and 20 MHz, respectively, before and after immersion for ten days in SBF solution using gold-plated stainless-steel electrodes in a parallel plate capacitor arrangement.

## Results and discussion

### Characterization of raw materials using XRD and HRTEM-SAED

**Figure 3:**
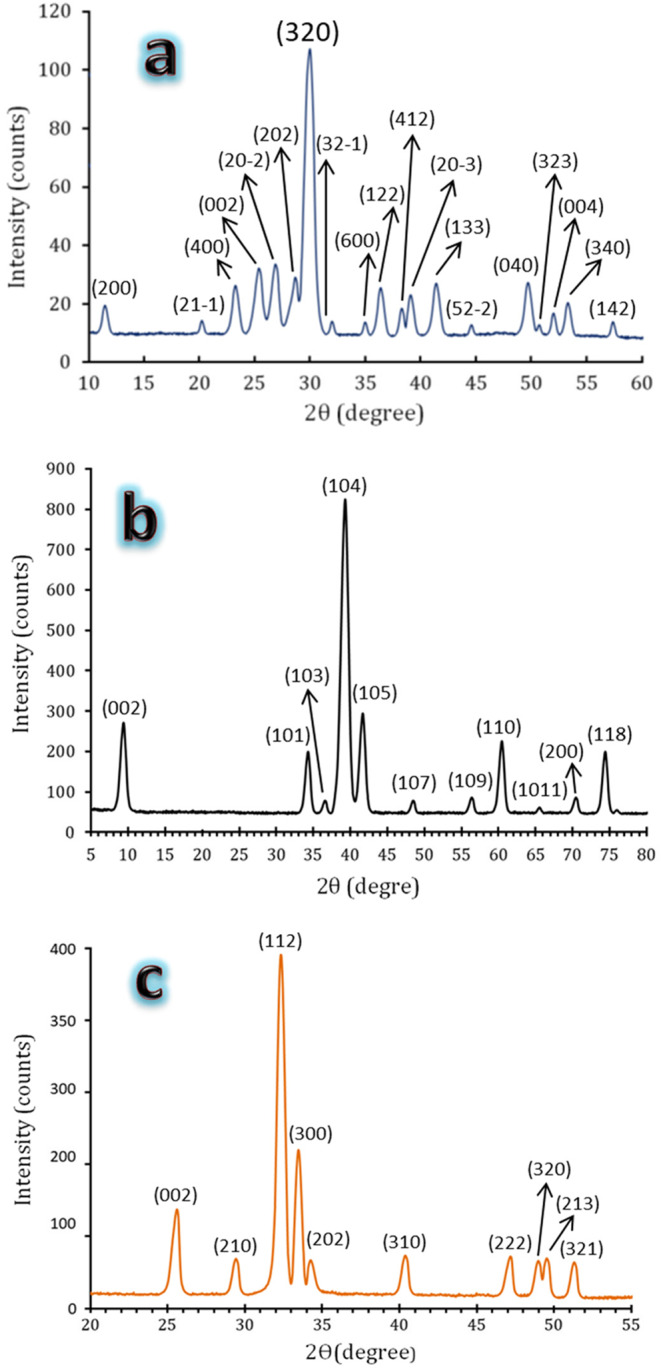
XRD patterns of as-prepared raw materials, i.e.,**a**) CaSiO_3_, **b**) HA, and **c**) Ti_3_AlC_2_ powders.

Figure 3(a–c) displays the structure of the initial materials‒CaSiO_3_, HA, and MAX phase powders, respectively‒that underwent XRD examination. Examining the patterns in Fig. 3b and c and comparing them to the ICCD file cards 19–0272 and 52–0875 clearly demonstrates the successful preparation of the HA and MAX phases, respectively. This is exemplified by the absence of peaks other than those forming the HA and MAX phases. Furthermore, Fig. 3a showcases the purity and absence of contaminants in the purchased CaSiO_3_ (84–0655) powders.

**Figure 4:**
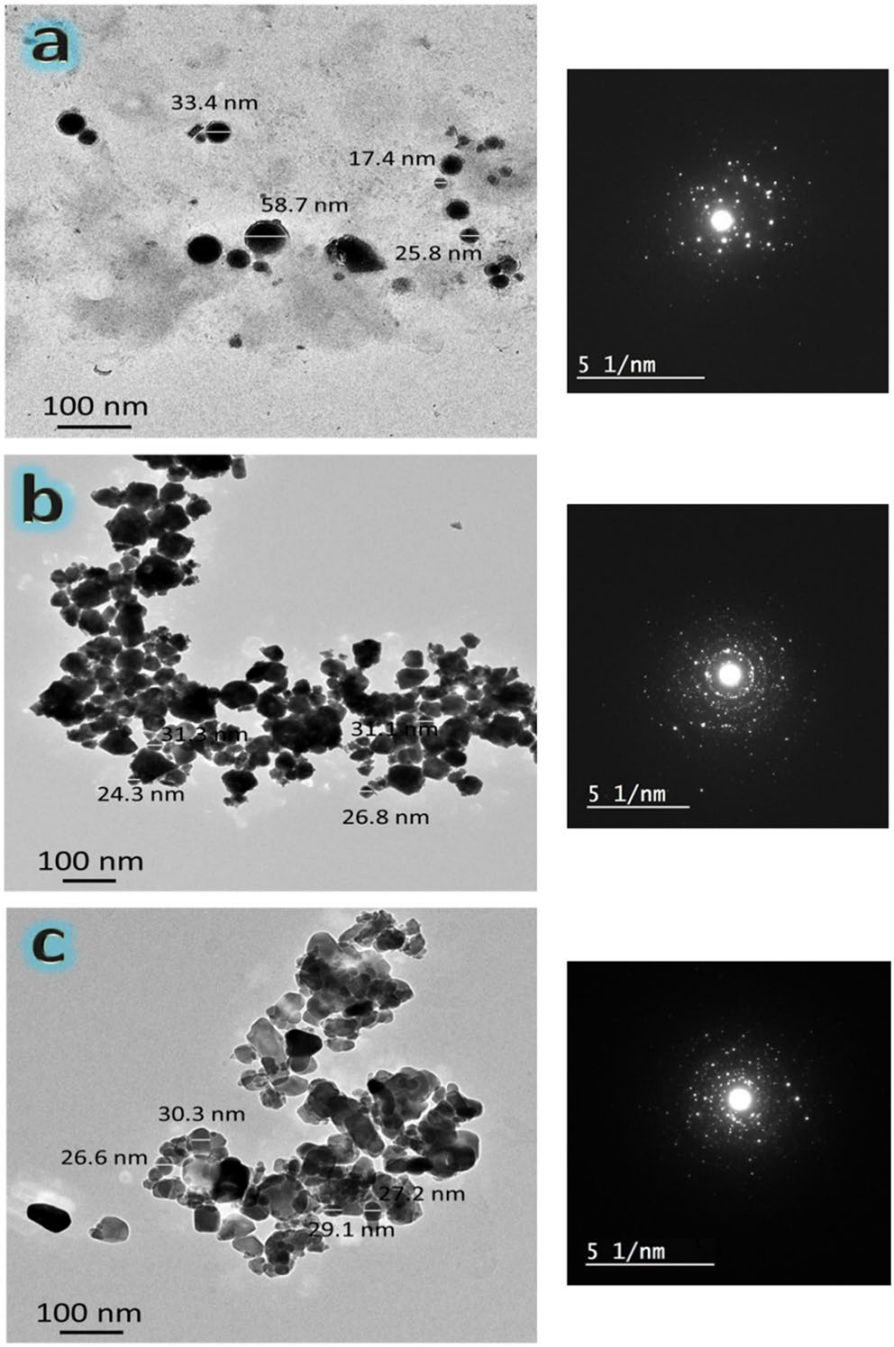
HRTEM images and the corresponding SEAD patterns of **a**) CaSiO_3_, **b**) HA, and **c**) Ti_3_AlC_2_ nanopowders.

Figure 4(a-c) shows the TEM images of CaSiO_3_, HA, and Ti_3_AlC_2_ powders in this order and their corresponding SAED patterns. Figure 2 also represents the average particle size of the raw materials used to prepare these samples, i.e., CaSiO_3_, HA, and Ti_3_AlC_2_. Upon closer examination of this image, it becomes clear that the raw materials for the study consist of spherical particles. The HA particles exhibit significant agglomeration because they were manufactured using mechanochemical synthesis. This aggregation appears less dense in the prepared MAX phase. This less aggregation is due to the MAX phase because it is a ceramic material with higher strength (brittle) compared to HA. Therefore, it acts as a grinding ball and undergoes fragmentation, which helps reduce the powder particles’ size, unlike HA particles, which have a lower strength, leading to clearer aggregation. The nanoscale range should include CaSiO3, HA, and Ti3AlC2 particles. 33.82, 28.37, and 28.30 nm particle sizes have been recorded for the CaSiO_3_, HA, and MAX phases, respectively. In addition, the SAED patterns obtained from the d-spaced ICCD file cards (84–0655), (19–0272), and (52–0875) indicate the existence of polycrystalline diffraction rings, confirming the crystalline nature of the raw materials utilized. These phases include the CaSiO_3_, HA, and MAX phases, which will be covered in more depth in Sect. 3.2.1.

### Characterization of the produced nanocomposites

#### Phase structure

**Figure 5:**
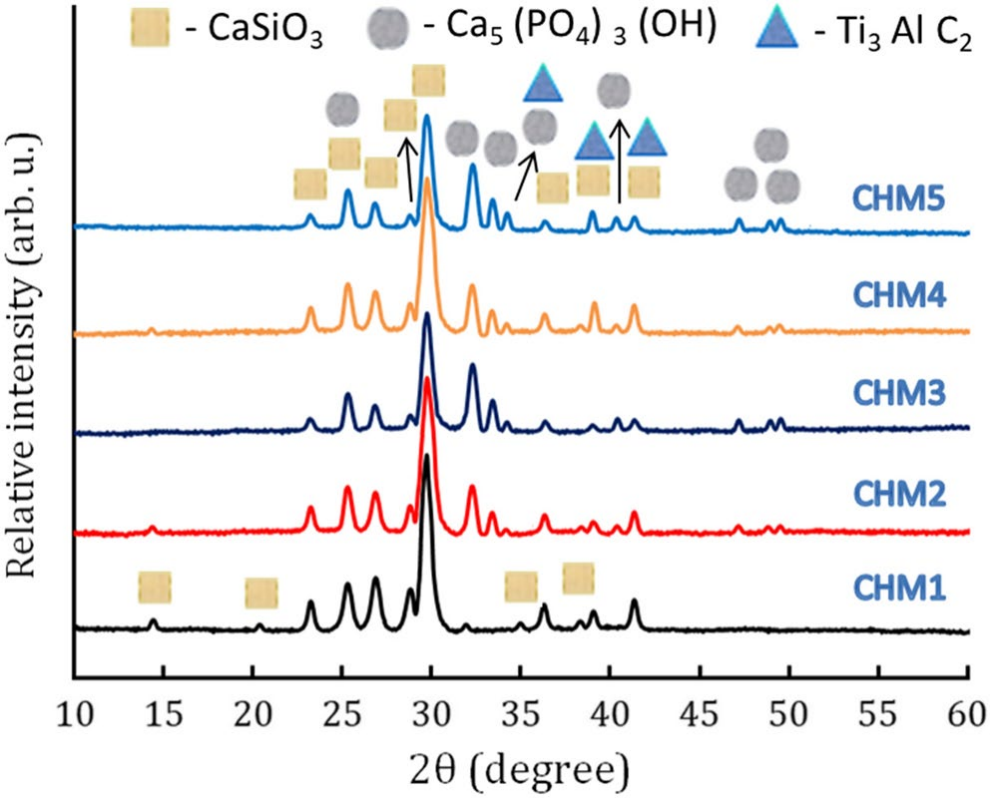
The XRD patterns of all samples after sintering at 1050 °C for one hour.

Figure 5 shows the phase composition of the powders generated and sintered at 1050 °C for one hour, as analyzed using the XRD technique. This figure indicates that the XRD pattern of sample CHM1 solely includes CaSiO_3_. However, the HA characteristic peaks are visible in the CHM2 sample because it is mixed with CaSiO_3_ at a volume of 20%. At the same time, the CaSiO_3_ characteristic peaks become less intense. The increase in HA content to 40% replicates the same situation in sample CHM3. As expected, the intensity of CaSiO_3_ peaks shows a further decrease.

Conversely, the CHM4 and CHM5 samples showed a minor contribution from the MAX phase, owing to its addition of 5 vol%. The peak intensities of CaSiO_3_ and HA in samples CHM4 and CHM5 remain unchanged due to the addition of the MAX phase, which did not replace the previous phases of CaSiO_3_ and HA. An essential indicator of the absence of contamination during the grinding and/or sintering procedures is the absence of other phases. It is critical to ensure that the nanocomposites are nanostructured materials by observing that all diffraction peaks are wider than the standard patterns of CaSiO_3_, HA, and MAX phases. Noteworthy nanostructured materials provide an advantage due to their enhanced interactions with proteins and bone cells, which is beneficial for biomedical researchers.

#### Analysis of the surface morphology

**Figure 6:**
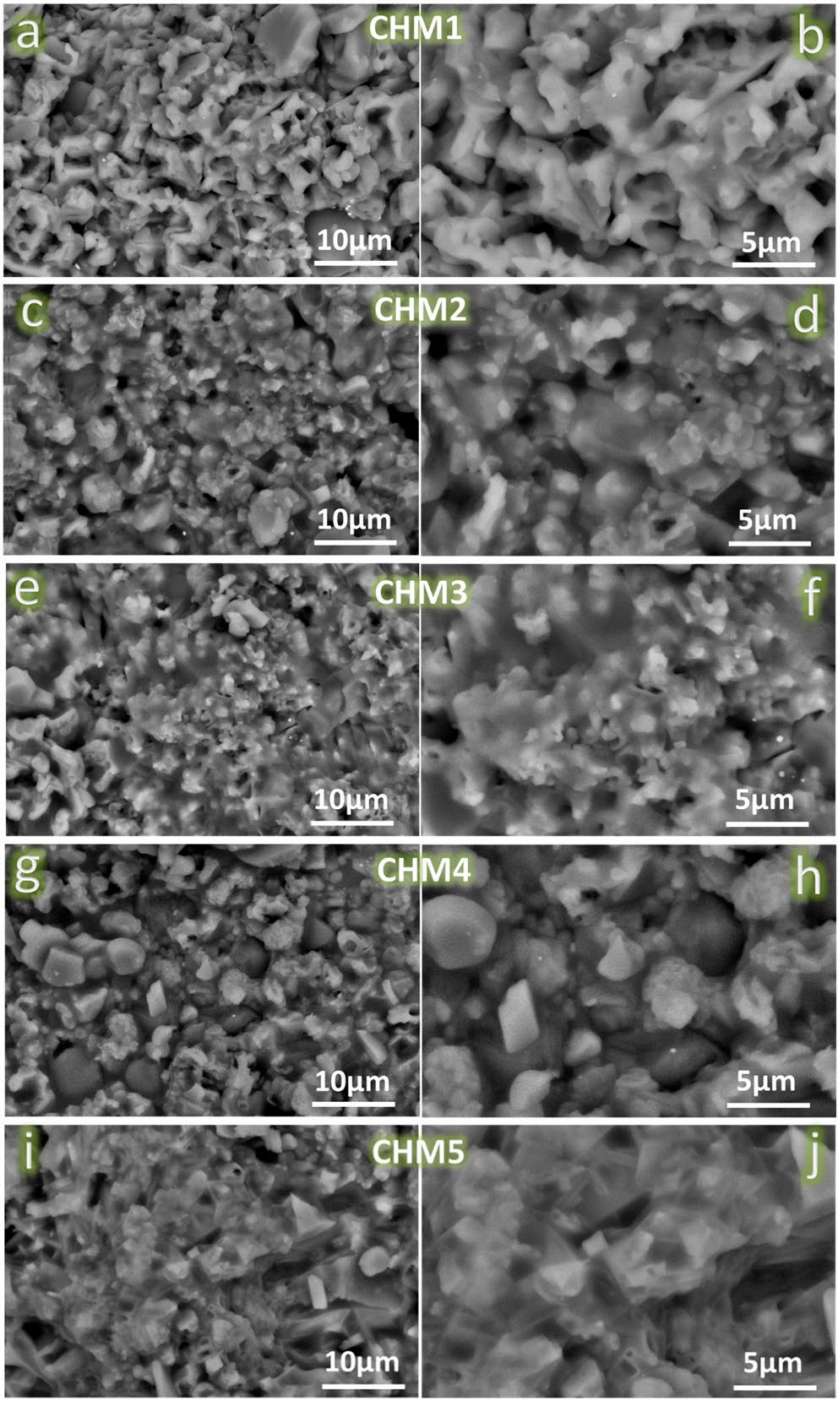
FESEM images, at two different magnifications, of CHM1 (**a**, **b**), CHM2 (**c**, **d**), CHM3 (**e**, **f**), CHM4 (**g**, **h**), and CHM5 (**i**, **j**) samples before soaking in SBF solution.

Figure 6(a˗j) displays the surface morphology of all samples sintered at 1050 ℃ for one hour, seen at two distinct magnification levels. This research used different magnification levels to determine the amount of condensation in sintered samples and observe the effects of adding HA and MAX phase contents to the CaSiO_3_. The FESEM analysis indicates that the selected sintering temperature is insufficient to achieve a fully dense structure in the CHM1 sample. Clear indications confirm this, including uniformly spaced tiny holes and grain enlargement. This densification is enhanced in the CHM2 and CHM3 samples. The reduction in the holes is due to the increasing concentration of nano-HA (20 and 40 vol%) in the samples, which helps seal the pores. Enhancing densification is another benefit of adding HA since it has a lower melting point than CaSiO_3_. The condensation is marginally reduced in the CHM4 and CHM5 samples because of the inclusion of the MAX phase, which requires a higher sintering temperature to exhibit favourable condensation properties. The densification was not significantly impacted due to the tiny MAX phase supply of the sintered samples.

### Biological features of the prepared nanocomposites

#### Bioactivity

**Figure 7:**
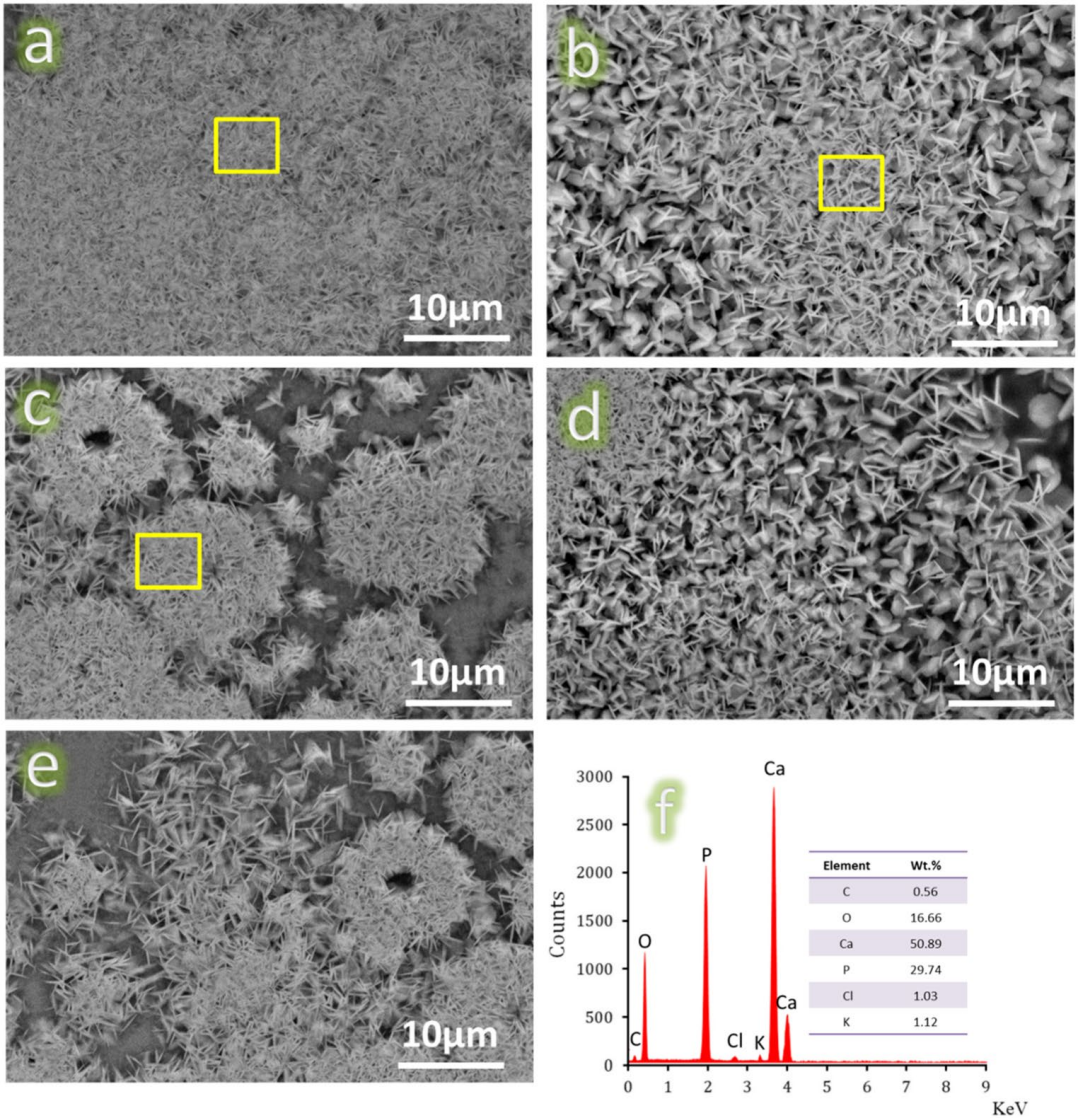
FESEM micrographs of (**a**) CHM1, (**b**) CHM2, (**c**) CHM3, (**d**) CHM4, and (**e**) CHM5 samples coupled with the EDX spectra of the formed HA layer on the surfaces of the CHM1, CHM2, and CHM3 samples after soaking in the SBF solution for ten days.

Biomaterial bioactivity is often assessed by its ability to form a bone-like HA layer (Ca_10_(PO_4_)_6_(OH)_2_) on its surface when exposed to an SBF solution. The technique is simple and cost-effective. The term “bioactive property” denotes a material’s ability to form the necessary layer. This indicates that the material should strongly adhere to the adjacent living bone tissue when implanted in the human body^25^. According to this concept, all sintered samples were incubated in SBF solution for ten days. The samples were analyzed using FESEM to visually demonstrate the formation of the apatite layer on their surfaces, as seen in Fig. 7(a-e). Figure 7a clearly shows the well-formed apatite layer on the surface of the CHM1 sample. This exceptional bioactivity may be elucidated by referencing Ref^26^. , which states that when samples containing CaSiO_3_ are placed in SBF, calcium (Ca^2+^) ions are exchanged with hydrogen (H^+^) ions in the solution, leading to the creation of silanol (Si-OH) groups on the sample surfaces. As a result, when the pH of the SBF increases near the immersed sample, the Si-O- groups on the surface become negatively charged. Due to these following events, Ca^2+^ ions are attracted to this negatively charged surface and form the desired layer, HA. The remarkable bioactive characteristics of CaSiO_3_ found align well with those reported in the literature^27,28^. However, the bioactivity behaviour is gradually decreasing in the CHM2 and CHM3 samples, as seen in Fig. 7b and c, due to the considerable decrease in the CaSiO_3_ content in these samples and successive increases in the HA content.

Compared to CaSiO_3_, HA’s bioactivity mechanism is a little bit different. An amorphous calcium phosphate (Ca-P) layer forms on the surface of HA due to interactions between its negatively charged (PO_4_)^3−^ and positive charges (Ca^2+^) in the SBF solution. As a result, HA interacts with (PO_4_)^3−^ in SBF to create deposits of bone-like apatite that crystallizes into calcium-deficient amorphous calcium phosphate and crystallizes on the surface of HA. This is confirmed by the appearance of dense colonies of HA crystals without complete coverage of the whole surface. According to the literature^29–31^, CaSiO_3_ possesses a higher bioactivity index than HA. As expected, since the addition of the Ti_3_AlC_2_ phase was not at the expense of other bioactive phases, i.e., CaSiO_3_ and HA, and was added in a small proportion, it did greatly affect the bioactivity behaviour of the examined samples, as deduced from Fig. 7d and e.

After immersing samples CHM1, CHM2, and CHM3 in SBF solution for ten days, we used the EDX technique to analyze their elemental composition as seen in Fig. 7f. The EDX spectra of the region enclosed in the yellow box may contain the following: Peaks corresponding to Ca, P, H, and O may be present. The ratio of Ca to P in all samples studied was about 1.71, indicating the formation of a non-stoichiometric HA layer on their surface. The C peak is also there because of a carbonated hydroxyapatite (CHA; Ca_10_(PO_4_)_6_(CO_3_)_6_(OH)_2_) layer and/or C in the MAX phase on the surface of the samples. This figure also shows that the CHA layer completely covers the distinct parts of the sample surface, as indicated by the absence of certain peaks such as Si, Al, and Ti. It is worth noting that small peaks of Cl^‒^ and K^+^ ions were observed due to traces on the surface of the samples from the SBF solution.

#### Biodegradability tests

**Figure 8:**
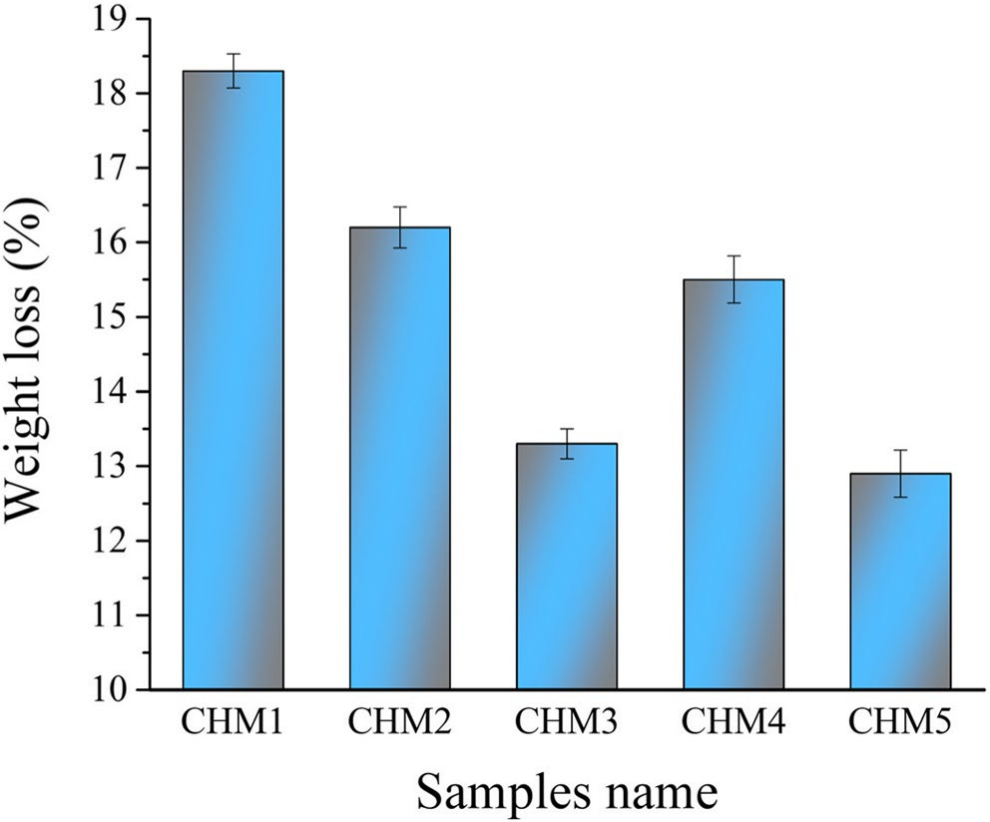
Percentage weight loss of all prepared samples after treatment with SBF solution for ten days.

The rate at which biologically active substances release ions has an evident influence on their bioactivity behaviour. Put another way, the surface reactions of the grains in the tested samples could lead to the forming of a Ca-P layer. These reactions result in the release of various elements, making the sample lighter. In this regard, weight loss measurement is a valuable technique for monitoring the kinetics of conversion and sample dissolution^32^. After immersion in the SBF solution for ten days, the weight loss percentages for each sample are shown in Fig. 8, along with the calculated mean value, variance, and standard deviation of the results obtained, which are tabulated in Table 2. The findings indicated that the CHM1 sample had a weight loss of around 18%—the proportion of weight loss in the CHM2 and CHM3 samples reduced when the HA concentration was increased. The Ti_3_AlC_2_ phase in the CHM4 and CHM5 samples considerably reduces the weight loss percentage. It should be mentioned that this work’s outcomes are better than the ones covered in earlier studies. Padmanabhann et al.^33^ examined the degradability of the 50 CaSiO_3_–50 HA scaffold by evaluating its weight loss after soaking in buffered Tris–HCl solution for different durations (1, 3, 7, 14, and 21 days) at 37 ℃. After seven days, it was revealed that the degradation rate of the 50 CaSiO_3_–50 HA scaffold reached around 25%. Another study conducted by Zhang et al.^34^ enhanced the biodegradability of CaSiO_3_ by adding three different materials, i.e., HA, strontium phosphate Sr_3_(PO_4_)_2_, and calcium sulfate (CaSO_4_.2H_2_O). Weight loss measured after ten days of soaking CaSiO_3_/10 HA/10 Sr_3_(PO_4_)_2_/15 CaSO_4_.2H_2_O (wt%) in SBF solution was about 15%.

**Table 2:** The mean value, variance, and standard deviation of weight loss occurring in samples after soaking in SBF solution for ten days.

Sample	Mean value	Variance	Standard deviation
CHM1	18.3	0.052	0.228
CHM2	16.2	0.076	0.276
CHM3	13.3	0.040	0.200
CHM4	15.5	0.100	0.316
CHM5	12.9	0.101	0.318

The controlled degradation of CaSiO_3_ can be explained by the very different solubility product constants (KSP) for CaSiO_3_ and HA. K_SP_ for CaSiO_3_ is 2.5 × 10^−8^, and K_SP_ for HA is 10^–53^. This implies that CaSiO_3_ dissolves in SBF more quickly than HA. The solution-mediated process is believed to be one of the fundamental mechanisms responsible for the material’s biodegradability^35,36^. Based on the above, it can be concluded that the MAX phase has a higher chemical stability than HA, leading to a decrease in weight loss. The following section, Sect. 3.4.1, will discuss how the successive addition of HA improved the densification behaviour, reducing diffusion and enhancing bonding between CaSiO_3_, HA, and MAX phases. It is well known that specific therapeutic applications, such as periodontal bone restorations, sinus lift treatments, spinal fusion, and fracture fixation, consider a relatively moderate deterioration rate acceptable^20^.

ICP-AES was used in this study for two primary purposes. Comparing the amounts of Ca^2+^ and P^3‒^ ions before and after soaking the samples in SBF solution for ten days is necessary to confirm the findings related to the bioactivity of the samples. To assess the amounts of Si^4+^, Ti^4+^, and Al^3+^ ions released from all samples after being submerged in SBF solution for ten days to ensure compliance with global safety standards, as listed in Table 3.

**Table 3:** Elemental concentrations in SBF solution before and after soaking all samples in SBF solution for ten days.

Sample code	Elemental concentrations (ppm)				
	**Ti** (± 0.002)	**Al** (± 0.002)	**Si** (± 0.2)	**Ca** (± 0.5)	**P** (± 0.2)
SBF	-----	-----	-----	100.2	30.9
CHM1	-----	-----	42.9	89.5	22.1
CHM2	-----	-----	32.1	84.3	19.5
CHM3	-----	-----	20.6	80.5	15.8
CHM4	0.01	0.02	28.3	81.4	17.4
CHM5	0.01	0.03	15.4	76.7	12.3

The concentrations of Ca^2+^ and P^3‒^ in the solution are decreasing consistently, as seen in the figure. The release of Ca^2+^ ions from CaSiO_3_ and HA in the samples causes an apatite layer on the surface. Additionally, the dissolution of CaSiO_3_ leads to an elevation in the concentration of Si^4+^ ions in the CHM1 sample. The Si^4+^ ion level decreases in the CHM2 and CHM3 samples owing to the presence of 20 and 40 vol% HA, which reduces these specimens’ degradation rate. The quantity of Si^4+^ ions is crucial in generating Si-OH groups and an apatite layer on the sample’s surface. The inclusion of 5 vol% of the MAX phase resulted in improved regulation of CaSiO_3_ release. The concentration of Ti^4+^ ions remained steady in the CHM4 and CHM5 samples analyzed, consistently measuring below 0.01 ppm. The findings of several academics support this seeming paradoxical conclusion.

The degradation of Ti depends on time, as mentioned in reference^37^. Another investigation confirmed the study’s findings^38^, indicating Ti’s slow depreciation. Therefore, the concentration of Ti^4+^ ions might rise in the SBF solution during an extended incubation period. Joseph et al.^39^. found that the TiAl_6_Nb_7_ plate did not release Ti^4+^ ions into the test liquids until 12 weeks of incubation. Mischler et al.^40^. asserted that the formation of the passive oxide layer influences biodegradation. The connection between the biological environment and the physicochemical, morphological, and mechanical features of the spontaneously growing Ti oxide coating is closely linked to the biocompatibility of Ti due to its resistance to deterioration (corrosion).

The findings indicated that most of the measured concentrations of the required components fall within the acceptable range. There is less toxicity data on the ingestion of aqueous Si^4+^ due to a scarcity of anecdotal reports of harm and an overall assumption of safety. Several studies on rats have shown that the No Observed Adverse Effects Level (NOAEL) for dietary Si is 50,000 ppm (mg/L)^41^. In North America and Europe, the typical daily intake of Si ranges from 20 to 50 mg. China and India had the lowest incidence of hip fractures due to their high daily intake of Si (140‒200 mg/day)^42^.

Typically, the concentration of Al in the blood is below ten mg/L or less than 60 mg/L for patients receiving dialysis. Values over 100 mg/L are considered toxic. Urine Al values below 55 µg/g of creatinine are deemed safe for people. A urinary Al level of 4 to 6 µmol/L indicates a threshold for neurological side effects, whereas a urine level of 100 *µ*g/L is considered the crucial concentration for developing neurological problems. Al poisoning may be evaluated by analyzing the concentration of Al in hair, nails, and sweat^43,44^.

### Analyzing samples properties

#### Physical properties of the investigated samples

**Figure 9:**
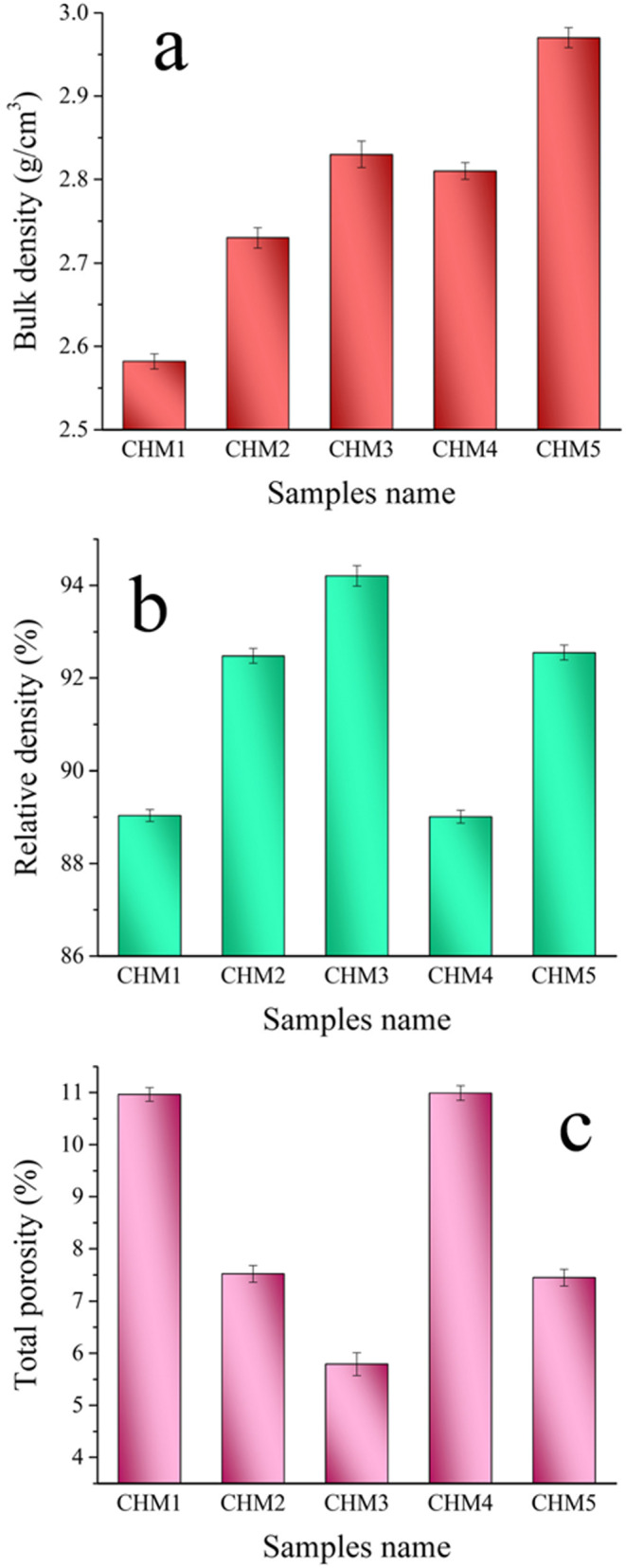
(**a**) Bulk density, (**b**) relative density, and (**c**) total porosity of all samples sintered at 1050 ℃ for one hour.

Figure 9(a-c) displays each sample’s bulk density, relative density, and total porosity. This figure shows that the bulk density of sintered samples depends on the percentages of HA and MAX phases added to CaSiO_3_. In other words, the bulk density of CHM2 and CHM3 samples increased significantly compared to the CHM1 sample due to the addition of HA by 20 and 40 vol%, respectively. The bulk density of CHM4 and CHM5 samples, compared to CHM2 and CHM3, continues to increase due to adding 5 vol% of the MAX phase to 20 and 40 vol% of HA, respectively. The bulk density improvement percentages for CHM2, CHM3, CHM4, and CHM5 samples compared to the CHM1 sample are 5.76, 9.23, 8.07, and 14.23%, respectively. The total porosity reduces in the CHM2 and CHM3 samples compared to the pure CaSiO_3_, i.e., the CHM1 sample. However, it increases in the CHM4 and CHM5 samples. The total porosity of the studied samples is 11.3, 7.9, 6, 11.1, and 7.8%.

The variability in the total porosity is due to nanoscale HA, which closes pores and improves the densification of the sintered samples. The MAX phase’s presence reduces the condensation behaviour of the sintered nanocomposites. The reduction in condensation is due to the MAX phase’s high melting point of 2100 °C compared to CaSiO_3_, which has a melting point of 1540 °C. Hence, the selected sintering temperature is inadequate for achieving a compact structure. Describing the sintering procedure is essential for the reader to clarify this vital feature.

In summary, effective densification depends on the selection of sintering temperature throughout the three stages of the sintering process, as mentioned in Ref^45^. Powder compressibility aids in initial contact formation. The particles form secondary bonds by creating “necks” that attach them. These necks form when the sintering temperature reaches about two-thirds of the melting point. When residual porosity is closed, the separated particles become completely bound and invisible. The results in Sect. 3.2.2 align well with the ones mentioned.

#### Mechanical properties

**Figure 10:**
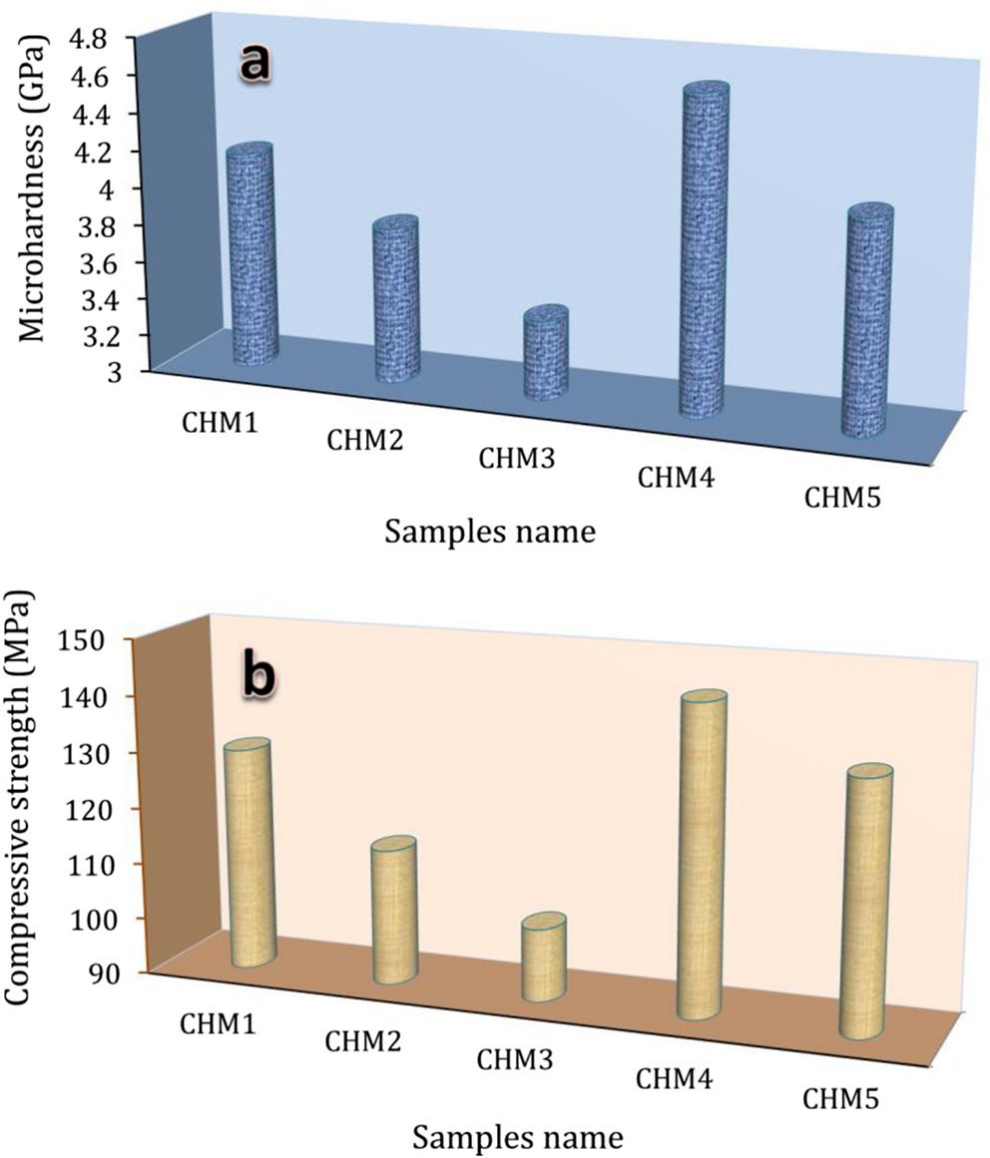
(**a**) Microhardness and (**b**) compressive strength of all samples sintered at 1050 °C for one hour.

The primary objective in creating nanocomposites for bone replacement is to have mechanical properties that resemble real human bone. Due to their significance, all sintered nanocomposites’ microhardness and compressive strength were tested and shown in Fig. 10(a, b). The microhardness of the tested samples decreases considerably with a sequential rise in HA concentration from 0 to 40 vol% and increases noticeably with the inclusion of the MAX phase. The calculated microhardness for CHM1, CHM2, CHM3, CHM4, and CHM5 samples are 4.2, 3.85, 3.3, 4.55, and 3.9 GPa, respectively. On the contrast side, Mehrali et al.^46^ improved the microhardness of CaSiO_3_ by successive addition of graphene nanoplatelets, i.e., 0.5, 1, 1.5, and 2 wt%, and they found that the obtained values were 6.38, 7.45, 5.58, and 4.60 GPa. Shirazi et al.^47^ and his colleagues attempted to improve the mechanical properties of CaSiO_3_ by adding 5 vol% of Al_2_O_3_ and obtaining that the microhardness increased from 6.2 GPa to 7.2 GPa. Papynov et al.^48^ used SPS technology to get a new CaSiO_3_-HA biocomposite ceramic reinforced with a Ti6Al4V alloy matrix. They found that the microhardness value was 640 HV (6.27 GPa) at the interface of the bioceramics and alloy. It should be noted that from the perspective of materials science, these results are considered outstanding. Still, from the perspective of biomaterials science, these results are considered harmful, as they indicate the possibility of fracture or separation of the implant due to a mismatch in the mechanical properties at the bone-implant interface.

The measured compressive strength of all tested samples showed a similar trend, as it decreased due to successive additions of HA up to 40 vol% and then improved with the addition of the MAX phase by five vol%. The calculated percentages of compressive strength for the CHM1, CHM2, CHM3, and CHM4 samples, compared to CHM1, are ‒12.21%, ‒19.84%, + 10.68%, and + 3.81%. Fortunately, the observed samples’ compressive strengths range from 131 to 145 MPa, comparable to cortical bone’s 100–150 MPa strength.

However, other researchers attempting to enhance the mechanical properties of CaSiO_3_ by using other materials did not provide encouraging findings. For instance, Sha et al.^49^ used polyethene glycol-modified graphene oxide, with different contents up to 1.2%, to improve the compressive strength of tricalcium silicate. They found that the maximum value they obtained was 14 MPa, which is a very low value. On the other hand, Mansoor and Dasharath^50^ gradually increased the concentrations of HA and titanium dioxide (TiO_2_) in CaSiO_3_. Consequently, the resultant nanocomposites’ compressive strength increased to 225 MPa. The latter results mean that if these samples were implanted in human bone, the surrounding bone would suffer a stress-shielding effect. According to Wolff’s principle, the stress shielding effect causes severe bone weakening and its lack of essential stimuli for the ongoing rebuilding process. Because the mechanical characteristics of the MAX phase vary significantly from those of CaSiO_3_ and HA, we can explain these oscillating findings.

#### Tribological properties

**Figure 11:**
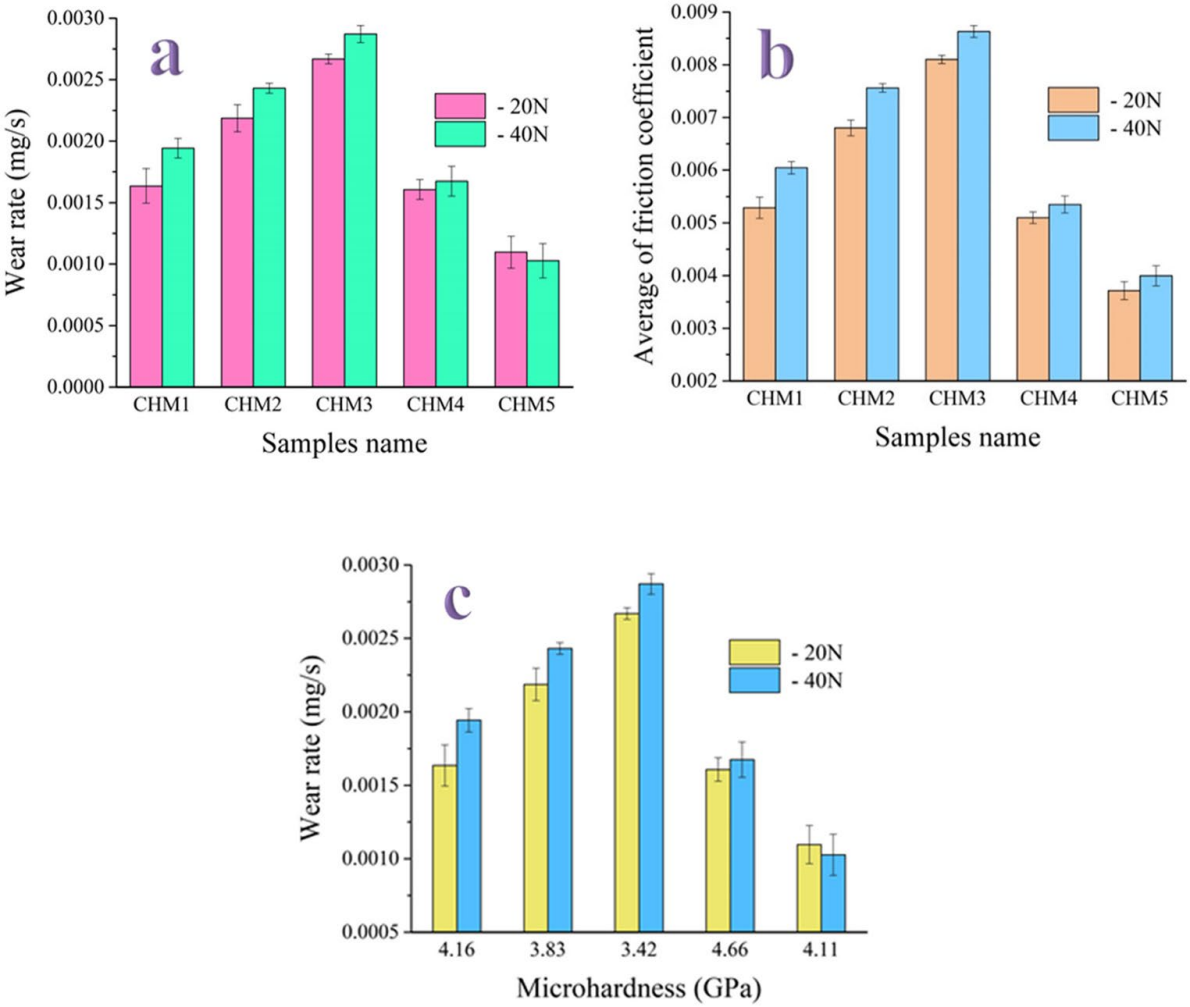
(**a**) Wear rate, (**b**) coefficient of friction, and (**c**) wear rate versus microhardness of all sintered samples at different applied loads, i.e., 20 and 40 N.

Since body fluids are the only lubricant surrounding an implant in a human bone, inadequate lubrication may result in a significant amount of wear debris, activating osteoclastic cells to dissolve the bone and loosen the implants. Given this crucial information, it is imperative to investigate the tribological properties of biomaterials for potential applications in the bone healing process^51^. In this regard, Fig. 11(a-c) illustrates the wear rate, the coefficient of friction, and the relationship between wear rate and microhardness for all studied samples under various loads. Additionally, Table 4 presents the weight loss and wear rate at different loads for all prepared samples. Based on the acquired data, it can be noticed that the wear rate of the sintered nanocomposites increased significantly with increasing HA concentrations (20 and 40 vol%), indicating more wear behaviour.

**Table 4:** Weight loss (mg) and wear rate (mg/s) at different applied loads, i.e., 20 and 40 N for all prepared samples.

Samples name	Weight loss (mg)		Wear rate (mg/s)	
	**20 N**	**40 N**	**20 N**	**40 N**
CHM1	0.9814	1.1658	0.0016	0.0019
CHM2	1.3120	1.4586	0.0022	0.0024
CHM3	1.6013	1.7222	0.0027	0.0029
CHM4	0.9643	1.0046	0.0016	0.0017
CHM5	0.6582	0.6164	0.0011	0.0010

On the other hand, an essential factor in improving the wear rate of CaSiO_3_/HA samples is the inclusion of the MAX phase. In other words, the inclusion of the MAX phase reduced the wear rate of the examined samples, unlike HA, which increased the wear rate. As expected, the coefficient of friction of the nanocomposites showed the same behaviour as before, as it improved with the addition of the MAX phase. At the same time, it increased as a result of the addition of HA. These encouraging results have several causes. First, as mentioned in Sect. 3.1, there is great value in preparing the MAX phase in the nanoscale range because the particles are bonded to one another, forming strong bonds between the CaSiO_3_, HA, and MAX phase particles^52^. Second, the wear was significantly reduced by the sintered nanocomposites meeting acceptable densification requirements (Sect. 3.4.1). Finally, the complex components in the sintered samples, such as Ti_3_AlC_2_ and reduced wear behaviour, are consistent with the results of the mechanical property analysis. This interpretation is firmly based on the data presented in Fig. 10, which shows the relationship between the wear rate and microhardness of all sintered specimens. Notably, this relationship was calculated according to the Archard equation:

4 $$\:Q=K\frac{W}{H}$$

where *Q* is the wear rate, *K* is a constant known as the wear coefficient, *W* is the applied load, and H is the sample’s microhardness.

#### Electrical and dielectric characteristics both before and after soaking in SBF solution

**Figure 12:**
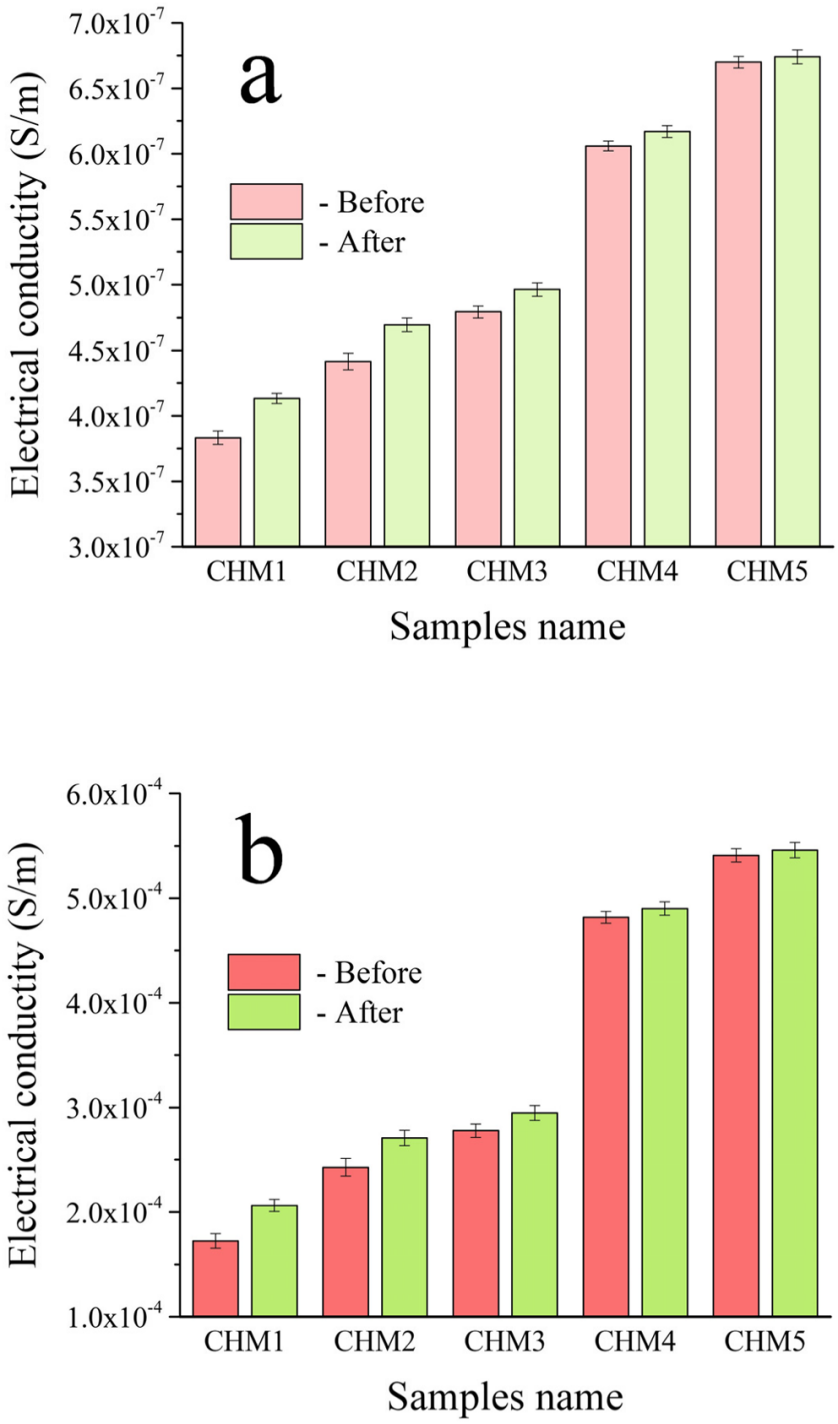
The AC electrical conductivity at **a**) lower frequency, i.e., 1 MHz, and **b**) higher frequency, i.e., 20 MHz, of all sintered samples measured before and after immersion in the SBF solution for ten days.

**Figure 13:**
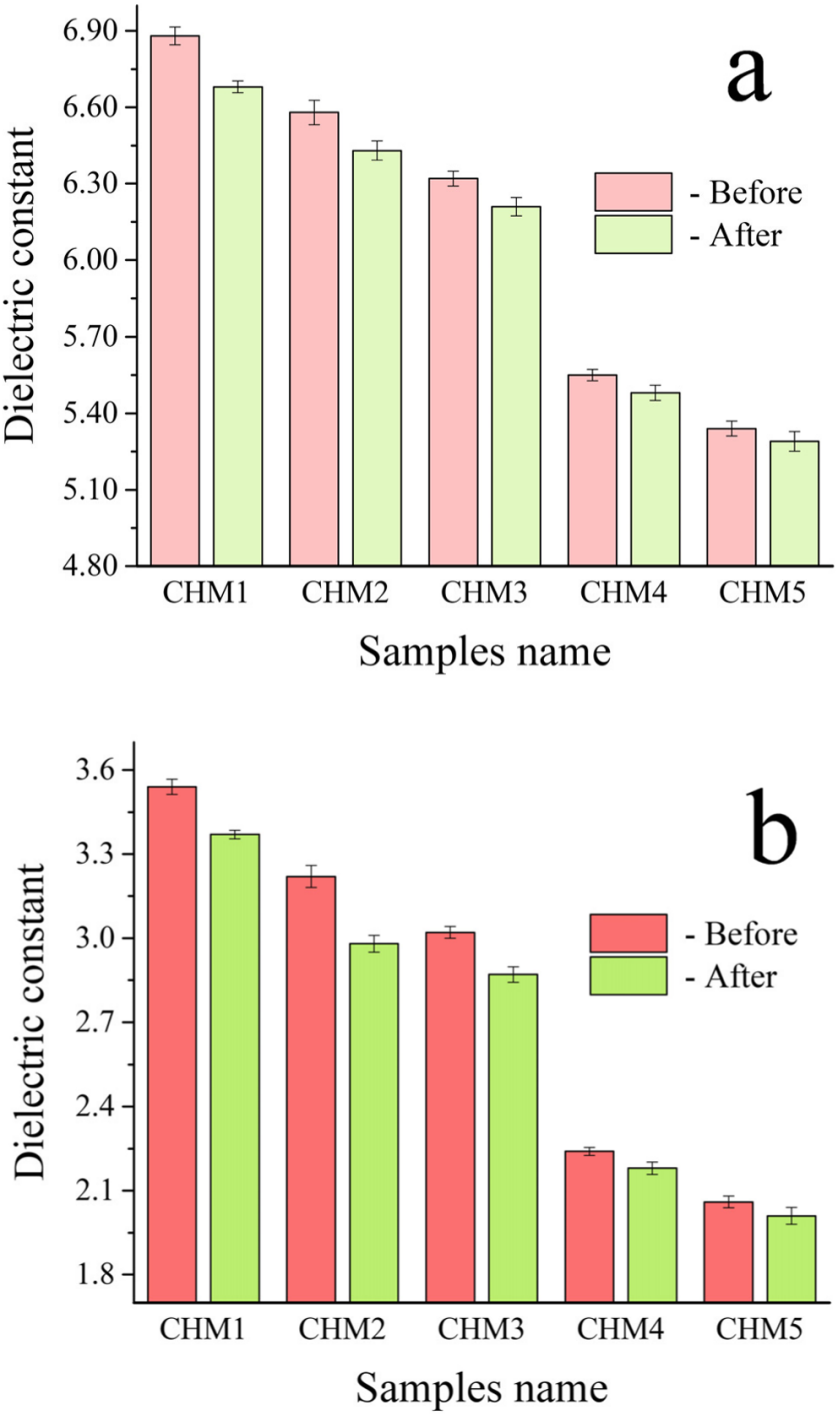
The dielectric constant at **a**) 1 MHz and **b**) 20 MHz of all sintered samples before and after soaking in the SBF solution for ten days.

**Figure 14:**
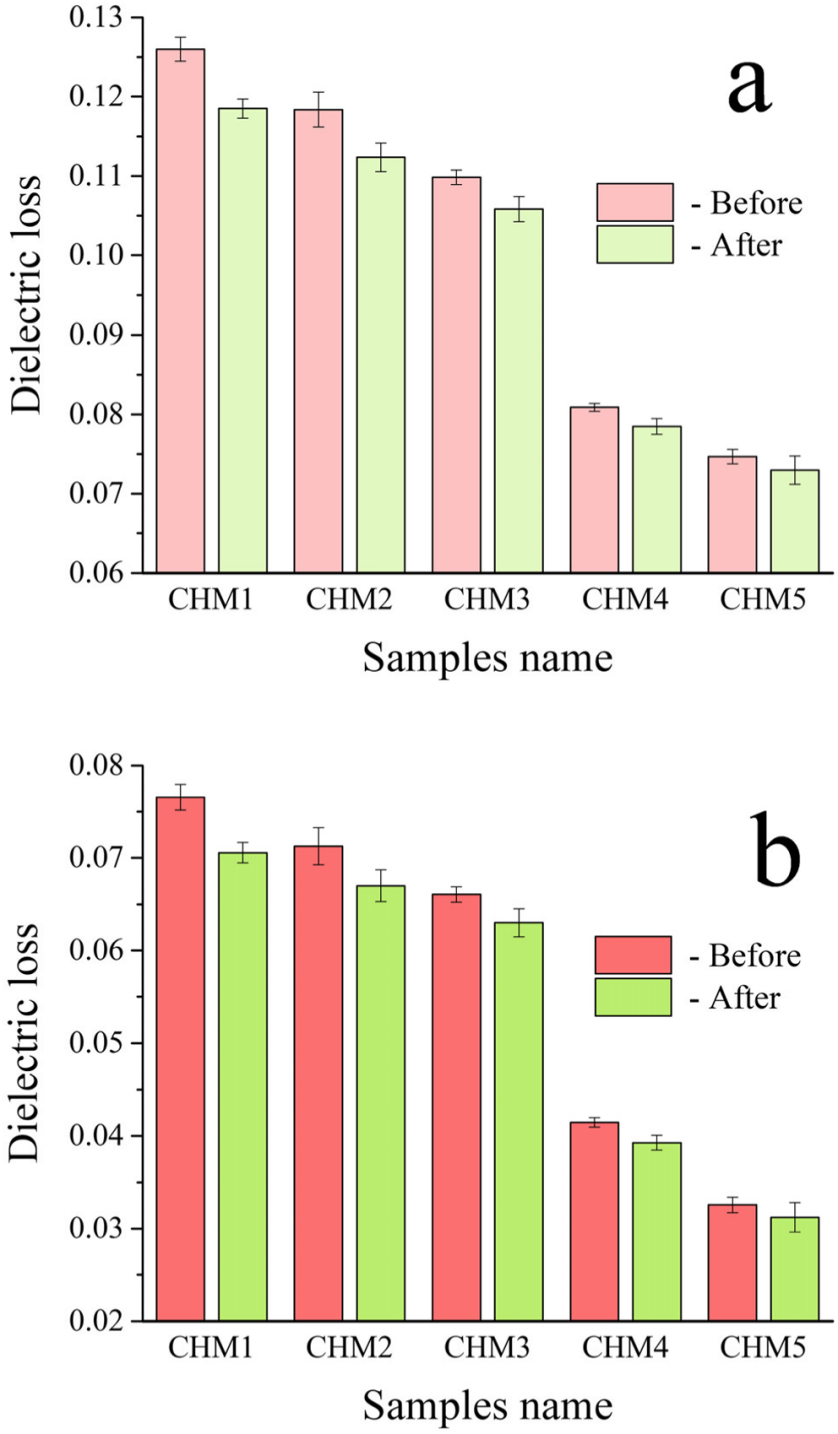
The dielectric loss at **a**) 1 MHz and **b**) 20 MHz of all sintered samples before and after soaking in the SBF solution for ten days.

Biomaterials with strong electrical characteristics have been shown to enhance bone development promotion significantly. It is crucial to analyze the electrical conductivity, ε′ and ε′′ at frequencies of 1 and 20 MHz for the samples obtained, as shown in Figs. 12, 13 and 14. Figure 12 shows that the AC conductivity increases with higher amounts of HA and MAX phase, as well as an increase in frequency from 1 MHz to 20 MHz. This discovery supports the understanding that several parameters, such as chemical composition, microstructure, porosity of sintered materials, and applied frequencies, affect electrical conductivity^53^. The main factors influencing the electrical conductivity of the studied nanocomposites are the increase in applied frequency from 1 to 20 MHz, the addition of HA and MAX phase as they are electrically conductive materials compared to CaSiO_3_, and the favourable condensation behaviour observed. This can be described as follows: (i) HA and MAX phase exhibit superior electrical conductivity compared to CaSiO_3_, (ii) Increasing the frequency to 20 MHz increases the mobility of charge carriers, enhancing the conduction mechanism in these samples^54^, and (iii) Obtaining a dense microstructure with reduced porosity is crucial for achieving optimal electrical conductivity. The reduction in porosity enhances electron transport. Due to the non-conductive nature of pores in sintered samples, free electrons encounter fewer pores along their route, leading to a reduction in electrical resistivity. The findings obtained are compared with those detailed in another source^55^. Equation (5) was used to demonstrate the material’s complex dielectric constant:

5 $$\varepsilon=\varepsilon'+j\ \varepsilon''$$

The term ε′ represents the real component of the dielectric constant, which quantifies the energy stored from the electric field and determines the orientation of dipoles in the material. Conversely, ε′′ represents the power dissipated in the dielectric and corresponds to the imaginary part of the dielectric constant, known as dielectric loss. In ceramic materials, charge carriers cannot travel freely without applying an alternating field to induce polarization. With increasing frequency, charge carriers move through the dielectric and become stuck at a defect site, creating an opposing charge that hinders their movement and reduces the ε′ value^56^. Thus, as seen in Fig. 13, the ε′ drops as the frequency increases. Figure 13 shows that the ε′ lowers as the HA and MAX phase concentrations increase. The decrease in ε′ as the quantities of HA and MAX phase increase may be attributed to their superior electrical characteristics compared to the CaSiO_3_ phase. In CHM4 and CHM5 samples, the drop in ε′ is somewhat offset by an increase in overall porosity%, as previously noted in Sect. 3.5.1, which reduces grain boundary connectivity. The frequency dependence of ε′′ is associated with conductance, ion relaxation, deformation, and vibration. Conductance and ion relaxation are the primary contributors to ε′′ at low frequencies. Meanwhile, the other two components become more noticeable at higher frequencies. Figure 14 shows that the ε′′ falls as the frequency, HA, and MAX phase contents increase. This is because of the reasons described above.

To investigate how the formation of a bone-like layer on the surface of the prepared samples affects their electrical and dielectric properties, the electrical conductivity, ε′, and ε′′ were measured at the same frequencies as before. The results are presented in Figs. 12, 13 and 14. The findings showed that the HA layer significantly enhances the AC conductivity of the CHM1 and CHM2 samples. The promising finding is due to the more excellent electrical conductivity of the apatite layer compared to pure CaSiO_3_ and CaSiO_3_ with 20 vol% of HA. The CHM3 sample exhibits a slight rise in the AC conductivity. The electrical conductivity of the CHM4 and CHM5 samples slightly increased despite the MAX phase having higher conductivity than the surface apatite layer. This formation of the apatite layer, which covers the porosity in the samples’ microstructure, is responsible for this increase in electrical conductivity. For the same reasons discussed earlier, the values of ε′ and ε′′ exhibited an opposite pattern as they decreased following incubation in SBF solution.

## Conclusions

Calcium silicate (CaSiO_3_) is a promising biomaterial for orthopaedic and dental applications. However, the implant’s quick deterioration and poor tribo-mechanical performance in the human body limit its long-term efficacy. In this context, nanocomposites consisting of CaSiO_3_, hydroxyapatite (HA), and titanium aluminium carbide (Ti_3_AlC_2_) MAX phases were produced using a powder metallurgy (PM) method to address the above challenges. The results demonstrated that the inclusion of HA and MAX phases notably enhanced the degradability of CaSiO_3_, as confirmed by weight loss measurements and inductively coupled plasma-atomic emission spectroscopy (ICP-AES). Fortunately, field emission electron microscopy (FESEM) shows minimal impact on the bioactivity of the resultant nanocomposites. However, the microhardness and compressive strength of CaSiO_3_ were decreased with the addition of HA but increased with the addition of 5 vol% of the MAX phase. Similarly, the presence of the MAX phase improved the tribological characteristics of CaSiO_3_. In addition, the produced materials showed improved electrical conductivity at frequencies of 1 and 20 MHz due to the presence of both HA and MAX phases. This result meant that the prepared samples had shown the ability to induce bone cell growth, making them intriguing for orthopaedic and dental applications.

## Data Availability

Data availability The datasets generated and/or analyzed during the current study are not publicly available because all data are presented in the article and therefore, there is no need to include raw data but they are available from the corresponding author upon reasonable request.
